# A Scoping Review of the Maternal Role at Older Age; Perceptions and Occupations

**DOI:** 10.3390/ijerph19010492

**Published:** 2022-01-03

**Authors:** Ruth Maman, Debbie Rand, Michal Avrech Bar

**Affiliations:** Department of Occupational Therapy, School of Health Professions, Sackler Faculty of Medicine, Tel Aviv University, Tel Aviv-Yafo 6997801, Israel; ruthmaman@mail.tau.ac.il (R.M.); drand@tauex.tau.ac.il (D.R.)

**Keywords:** mothers, roles, older women, occupation

## Abstract

Motherhood is a meaningful life role among adult women. Occupations within the maternal role of younger mothers have been well documented, but less is known regarding the maternal-role at older age. This review aimed to describe the occupations, activities, and perceptions that older women ascribe to their maternal role. In the future, this information may promote health and wellbeing of older women. A systematic search of peer reviewed articles, that included healthy, community-dwelling mothers, 60 years of age or older, was conducted. Maternal-role occupations and perceptions of older mothers were identified and classified according to the Occupational Therapy Practice Framework (OTPF). Fourteen articles, representing 3102 older mothers, were included. The identified occupations and activities within the maternal role were from two categories: Instrumental Activities of Daily Living (IADL) (such as assistance with daily chores) and social participation (such as sharing holiday rituals). Three themes reflecting maternal-role perceptions were identified: providing support; relationship with children; and motherhood as a never-ending role. Maternal occupations were identified in only a few articles and from only two categories, IADL and social participation. These findings together with the perception that motherhood is a ‘never-ending’ role suggests that further research is needed to better characterize the maternal role of older women from an occupational perspective.

## 1. Introduction

As life expectancy continues to grow, the importance of identifying factors contributing to healthy aging increases [1]. Engagement in meaningful occupations, specifically among older adults, has been positively associated with health and wellbeing [2,3,4]. Stav et al. [5], in their systematic review of 59 longitudinal studies, also concluded that the participation of older adults in meaningful occupations, such as leisure, social, and religious activities, is related to positive health outcomes. Evidence in this field, led the World Health Organization to highlight the importance of researching the subjective view point of older adults regarding meaningful occupations, and incorporating this knowledge in health programs aiming to better promote participation [6].

Occupations are sets of activities (such as eating or shopping), carried out in various everyday contexts that people need, want, or are expected to do [7]. The Occupational Therapy Practice Framework (OTPF) defines eight main occupations: Basic and Instrumental Activities of Daily Living (BADL and IADL), social participation, leisure, rest and sleep, education, work, and play. Multiple activities can be involved in the execution of certain occupations; and multiple occupations can be involved in the process of fulfilling meaningful life roles [8].

Motherhood is one of the most common and desired life roles among adult women [9,10]. Mothers frequently identify themselves through motherhood, perceive the maternal role of higher importance and prioritize it over other life roles, such as work or community roles [11,12]. The maternal role is experienced by mothers as extremely meaningful and it includes various occupations and activities, such as caring and assisting children with IADL occupations [13]. Mothering occupations, which vary between woman, are largely influenced by social, cultural, and temporal factors [14,15]. Engagement in occupations within the maternal role interacts with mothers’ personal values and perspectives. For example, a mother can perceive feeding, bathing, and dressing a child as caregiving, but can also perceive these occupations as opportunities for teaching knowledge or passing on values to her child [16,17]. Personal values ascribe meaning to occupations within the maternal role, including occupations that are experienced as demeaning or less enjoyable, for example, getting children ready for school, arguing with children, or cleaning up their room. The value of being “a good mother” is reflected in mother’s perception that these occupations are important and necessary although sometimes undesired [18]. Sethi [17], discussed a developmental component that is embedded in the maternal role; mothers constantly evolve and mature in the mothering process, as role experience accumulates and as children grow older. Findings from a qualitative study of young mothers support this idea; when comparing two age groups of young mothers, the older mothers expressed less desire for perfection and less guilt regarding the maternal role as opposed to the younger mothers [16].

Despite the recognition of its chronologically developing nature, there seems to be a gap in the research of the maternal role at later stages of women’s lives [19]. Our literature search revealed only a few studies that included information regarding the maternal role occupations of healthy older women. Most studies focused on mothers with functional disabilities or mothers to children with disabilities [20,21,22,23]. Older women comprise a large and growing proportion of the older population, and face higher risks of illness and chronic conditions than older men [24,25]. Therefore, it is important to understand the maternal role as a meaningful life role that may motivate older women to participate in daily activities and occupations, and hence maintain or improve their health and wellbeing. The aim of this scoping review is to systematically explore the maternal role of healthy older women. We defined two research questions: (1) What occupations and activities are included in the maternal role among healthy older women? (2) What are the attitudes and perceptions of older mothers regarding mothering occupations at older age? These findings can be used in the future by health professionals, and specifically by occupational therapist practitioners, to enhance participation to help preserve the health and wellbeing of older women.

## 2. Materials and Methods

We conducted a scoping review, since this methodology is appropriate when conducting a knowledge synthesis of key concepts within an area of diverse evidence [26]. We followed the methodological framework of Arksey and O’Malley [27] which instructs five sequential steps: (i) Identifying research question; (ii) Identifying relevant studies; (iii) Study selection; (iv) Charting data; and (v) Collating, summarizing, and reporting results. We were additionally guided by the Preferred Reporting Items for Systematic Reviews and Meta-Analyses extension for Scoping reviews (PRISMA-ScR) [28] (see Appendix A).

### 2.1. Eligibility Criteria (Study Selection)

Peer-reviewed, full-text articles that were published in English were included in this scoping review according to the following inclusion criteria. The search was not limited by year of publication. Articles were eligible if they included community dwelling older mothers of healthy children. Mothers were 60 years of age or older (or at least some of the sample was 60 or over) and were independent in daily living. Studies that included only young mothers, or mothers with chronic conditions or disabilities or mothers of children with disabilities were excluded from this review.

### 2.2. Information Sources and Search Strategy (Identifying Relevant Studies)

A search strategy was developed by the research team in consultation with a certified librarian, following the PCC mnemonic (Population, Concept, and Context), recommended by the Joanna Briggs Institute (JBI) guidance for scoping reviews [29]. The search terms were: mothers, mothering, aged OR aging OR age, older, old, adult children, geriatric, and life changes. The search process was conducted using three databases: Medline, PsycINFO, and CINHAL.

### 2.3. Data Items and Data Collection Process

The data collection process was guided by recommendations of Levac [30] and included research team meetings to define the inclusion and exclusion criteria. Two independent raters reviewed the articles in two stages: titles and abstracts were reviewed first, then the relevant full texts were reviewed. Disagreements regarding inclusion of the articles were reviewed by two other raters, to make the final decision.

### 2.4. Charting the Data

The following data were extracted from the articles (a) author(s), (b) year of publication and country, (c) study question(s) or aim of the study, (d) participants, (e), study method and tools. In addition, the extracted data were examined for relevant information regarding occupations and perceptions related to the maternal role, according to the research questions. Maternal-role occupations and perceptions were then classified and organized according to OTPF: Domain and process 3rd Edition [8]. The occupations and perceptions that were identified fit the definition of IADL and Social Participation. Discrepancies regarding categorization were discussed between all co-authors until agreement was reached.

## 3. Results

**Figure 1:**
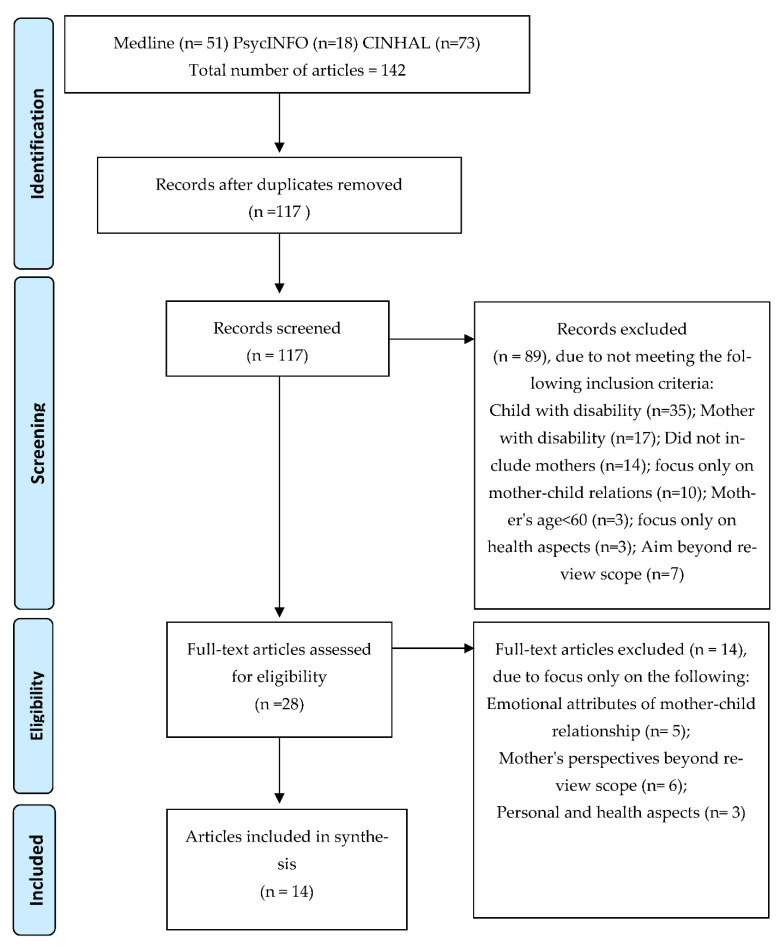
PRISMA diagram presenting systematic search process.

The systematic search yielded 142 articles, 117 after removal of duplicates and 89 records were excluded following the screening process due to not meeting the inclusion criteria (see Figure 1 PRISMA diagram). After the full-text of 28 articles were assessed for eligibility, 14 articles representing 3102 older mothers were included in this scoping review. These articles described studies which utilized qualitative (*n* = 5), quantitative (*n* = 8), and mixed method (*n* = 1) research methodology. All qualitative studies used interviews as the main research tool, mostly semi-structured, and one study conducted focus groups as well. The quantitative studies used self-report scales for assessing research measures (such as closeness in mother–child relationship or maternal support).

Six of the fourteen studies included only older mothers, the remaining studies included younger mothers as well, ranging from mothers aged 32 to 89 years old. The articles included were conducted by researchers from various fields: sociology (*n* = 5), social work (*n* = 3), gerontology (*n* = 2), psychology (*n* = 2), public health (*n* = 1), and occupational therapy (*n* = 1), and originated worldwide: North America (*n* = 8), Europe (*n* = 4), and Asia (*n* = 3) (see Table 1). As can be seen in Table 1, samples of the 14 articles included older mothers (*n* = 14 mothers to 803), to investigate varied study aims.

The maternal role occupations and perceptions that were identified in the articles matched the definitions of IADL and social participation occupations. IADL occupations are activities that support daily life within the home and community, including health and financial management and the care of others. Social participation occupations refer to activities that support the engagement in social situations and contexts, including in the family or the community [8].

### 3.1. Occupations within the Maternal Role of Older Women

IADL occupations within the maternal role were identified in 12 of the 14 articles. The focus in these studies was on the support older mothers give their adult children [32,34,36,38,39,40,41,42,43]. The IADL occupations included assisting children with cooking or shopping, running errands for the children, tending their home, providing them with financial support, and caring for the grandchildren. Other studies described exchanged support, which relates to support given by the adult children to their older mothers [31,33,35]; for example, finding a place for the mothers to live and taking care of the mothers when they were ill.

Social participation occupations within the maternal role were identified in eight articles. These studies highlighted social events and activities in which older mothers engage together with their children. Mothers and their children, participated in fun and enjoyable activities such as shopping, travelling, and gift giving. They visit their children and share holiday rituals together. Mothers described being in contact frequently with their children, sharing thoughts and opinions, and being involved in their children’s lives [31,32,33,35,37,38,40,44]

### 3.2. Perceptions and Attitudes of Older Women toward the Maternal Role

Three themes were identified in the articles reflecting mothers’ perceptions and attitudes towards the maternal role and are described below and in appear in Table 2. Theme I. Perceptions of Providing support were identified in seven articles. These articles described the value the mothers ascribed to supporting their children; for example, feeling rewarded by providing support to children. The mothers’ perceptions towards receiving support (exchanged support) were perceived as ambivalent, not all mothers felt comfortable with support given to them by their children [31,35,36,40,42,43]. Theme II. Perceptions of having a relationship with their children emerged from five articles. Close relationships with children are highly valued by mothers, sometimes perceived to reconcile the hardship of earlier stages of motherhood [32,33,38]. Closeness is also described as a complex attribute of the relationship between older mothers and their children; for example, Suitor et al. [37] discussed the phenomenon of mothers’ differentiation of closeness among their children and factors that contribute to it. Blieszner et al. [33], described older mothers’ experiences of a relationship with their daughters, which had positive aspects of closeness and companionship, but also aspects of conflict, complexity, and ambiguity; The mother’s relationship with her own child is also described to be extended to their spouse (children-in-law) [44]. Theme III. Motherhood as a never-ending role was discussed in two articles. Mothers described feeling responsible and accountable for upbringing of the children, the child’s growth, and success throughout life. The development of the maternal role over time is also demonstrated by the extension of the perceived responsibility towards the daughter/son in-law, as well as to the grandchildren. Mothers reported perceiving increased importance of the maternal role as the years go by, as opposed to other life roles (such as professional roles) that are perceived less as important over time [34,38].

## 4. Discussion

The goal of this review was to explore the daily occupations and perceptions associated with older women’s maternal role. We identified two categories of occupations within the maternal role: IADL and social participation, as well as three themes reflecting perceptions regarding the maternal role: providing support; relationship with children; and motherhood as a never-ending role. A certain symmetry is apparent between the identified occupations and perceptions: IADL occupations, which include caring for others, correspond with perceptions of providing support to children; and social participation occupations correspond with mothers’ perception of the relationships with their children, which naturally occurs in social contexts. This may suggest an interweaving dynamic interaction between mothers’ values and their daily occupations. Personal values reflect one’s goals or desires and include beliefs about what is good, correct, or important to do [45]. The way people choose which daily occupations they engage in, and the way they experience their engagement, is largely based on their personal values [46]. The perception that providing support is an essential part of motherhood, may reflect the value of ‘giving’, which has been previously associated with the maternal role of younger women [18,47]. This value may lead mothers to engage in the wide range of IADL occupations, which provides support to their children. However, since personal values are strongly affected by social contexts and cultural factors [48], it is expected that the choices of occupations and engagement within the maternal role will vary according to individual, social, and cultural factors. Schwarz et al. [39] for example, described the traditional Chinese obligation of adult children to support their older parents. Therefore, an older Chinese mother who identifies with this value may appreciate the receiving of support from her adult child, while the option of providing support to the child may be perceived negatively in relation to fulfilling her maternal role. Similarly, the identified value of having a close relationship with children may lead older mothers to engage in the social participation occupations that were identified in the reviewed articles.

The importance of understanding values of older adults, is in accordance with the recent global movement towards healthy aging. Enabling older adults to do what they value, supports current definitions of healthy aging, which expands the concept of health beyond traditional medical measures [6,49,50]. Participation, which means involvement in life situations, is the ability of older adults to perform daily activities and fulfill meaningful life roles. Participation is considered an important indicator of human health and well-being, therefore maintaining the participation of older adults and specifically of older women, is of paramount importance. According to the WHO’s International Classification of Functioning and Disability (ICF) model, participation is the consequence of interactions between an individual’s health status, personal, and environmental contextual factors [51]. Social participation of older adults has been found to be positively associated with self-rated physical and mental health [52,53]. Occupations of social participation were identified in eight of the reviewed articles, demonstrating the importance of social participation as part of the maternal role of older women. Participation in activities that contribute to others has also been found to be associated with enhanced outcomes of health and wellbeing of older adults [54,55,56]. For example, Forssen and Carlstedt [38] described the impact of close relationships with children and grandchildren on the perceived health of older mothers, which was expressed by feelings of self-worth that helped to endure pain, and hence increase their participation. Schwarz et al. [39], reported associations between support provided to children and the older mothers’ life-satisfaction. This suggests that the maternal role, as a meaningful life role, holds the potential to motivate older women, who are at risk of illness and chronic conditions [25,57], to participate in meaningful occupations, and hence achieve better health outcomes. Since the meaning that a person attributes to participation in activities is subjective and often covert [58], identifying the values and perspectives that may lead mothers to make personal choices regarding the engagement in these occupations, is important. 

This scoping review highlights the gap in the research of motherhood in older age, specifically research from an occupational perspective. Despite our wide search of year of publication, only fourteen eligible articles were identified and only five of them were published within the last ten years. Only one study specifically aimed to explore maternal occupations of older women, while the other studies partially reported on this topic within their original aims. In addition, only two maternal role occupations of the eight occupations defined by the OTPF, were identified in the reviewed articles. This possibly reflects paucity in the research of the maternal role at older age, rather than restricted participation in occupations of older mothers. Occupations such as leisure or BADL were not described in the articles and should be further researched.

### Limitations and Strengths

This is the first scoping review that aimed to identify the occupations and daily activities within the maternal role of older women. The articles included in the review were elicited from a wide range of research fields and were examined through the unique occupational perspective, using the OTPF [8]. Three databases were used for the searching process, while focusing exclusively on peer reviewed articles published in English language. It is possible that by using additional databases, by using alternative search terms or inclusion criteria other search results would have been found. The limited number of articles included in this review calls for a cautious interpretation of conclusions.

## 5. Conclusions

The scarce literature found in this scoping review supports the perception that being a mother is a meaningful and never-ending life role. Further research is needed in order to characterize the maternal role of older women, specifically through an occupational perspective. This information may be used to promote the health and wellbeing of older mothers, by enhancing their participation in meaningful occupations and life roles.

## Figures and Tables

**Table 1:** Description of the 14 articles included in review.

First Author and Year of Publication	Study Purpose	Discipline/ Country	Study Methodology/Tools	Participants
Bromberg 1983 [31]	To determine whether quality of mother–daughter relationship is related to mutual support given with respect to support given by daughters to mothers.	Social Work/United States	Quantitative	Older mothers (*n* = 75), widowed, Jewish. Age 65 and up
Henwood 1993 [32]	To investigate experiences, identities and relationships of mothers and daughters in a family life framework	Social Sciences/United Kingdom	Qualitative/semi structured interviews	Mothers (*n* = 60) aged > 50, who have adult daughters (age-up to early 40s)
Blieszner 1996 [33]	To explore relationships of older mothers and daughters, identify predictors of relationship quality and describe satisfactions and complaints related to the relationships	Gerontology/United States	Mixed methods/Structured interviews including 11 indicators of relationship interaction and quality and nine indicators of parent-child demographic characteristics	Older mothers (*n* = 15). Mean age 72, age range 66–85
Francis-Connolly 1998 [34]	To examine the mothering occupation and experience across life course	Occupational Therapy/USA	Qualitative/Semi structured interviews and focus groups	Mothers (*n* = 17), aged 32–83 with 1–8 children
Martini 2003 [35]	To explore the ways in which support from daughters to mothers is initiated and given and the types of support given from the perspective of mothers, and to explore the perspective of daughters in relation to their mothers’ feelings	Psychology/Canada	Quantitative	Older mothers (*n* = 43), age range 60–89, mean age 73. Adult daughters (*n* = 43) age range 29–67, mean age 44.
Suitor 2006 [36]	To explore the extent of within-family differences in older mothers’ support to children, and explain differences using factors drawn from between-family designed studies.	Sociology/United States	Quantitative	Older mothers (*n* = 556). Age range 65–78 (mean age—70.9; SD—3.1)
Suitor 2007 [37]	To identify factors that distinguish between mothers who report having a favorite child and mothers that report equal preference among all their children	Sociology/ United States	Quantitative	Older mothers (*n* = 566), age range 65–75 (mean age 70.9; SD—3.1).
Forssen 2008 [38]	To describe the mothering work and mothers’ perceived important conditions for doing it. To examine relations between mothering work and perceived health across mothers’ life course	Public Health/Sweden	Qualitative/Open interviews	Older Swedish mothers (*n* = 20). Age range 63–83
Schwarz 2010 [39]	To explore and compare relations between mothers’ life satisfaction and support provided to adult children among Chinese, Indonesian, and German women.	Psychology/China Indonesia Germany	Quantitative/Structured interviews	Older mothers (*n* = 406). mean age of rural Chinese mothers—64.69, urban Chinese mothers—68.04, rural Indonesian mothers—58.00, urban Indonesian mothers—67.16, German elderly mothers—69.57.
Schwarts 2015 [40]	To describe mothers’ experience of maternal role to co-residing adult children who have returned to live at parents’ home.	Social Work/Israel	Qualitative/In-depth, semi-structured interviews	Mothers (*n* = 14). Aged 58–74 with adult children (aged 30–40) who returned to live at home.
To 2015 [41]	To examine patterns of mother’s support distribution to their sons and daughters-in-law, the factors affecting them and changes over time	Sociology/China	Qualitative/Semi structured interviews	Older mothers (*n* = 34) aged 49–76. Married, including co-residing mothers- and daughters-in-law.
Lee 2017 [42]	To understand intergenerational financial transfers and subjective well-being of older mothers	Sociology/ South Korea	Quantitative/General life satisfaction estimation on a 0–100 scale; structured interviews—intergenerational economic exchange questions	Mothers (*n* = 803), with at least one married child living separately, age > 45, married mean age 57, widowed mean age 68.9
Bangerter 2018 [43]	To examine support given in context of life problems as perceived by mothers and their middle-aged children	Gerontology/United States	Quantitative/Frequency of support given-the Intergenerational Support Scale (ISS; Fingerman et al., 2009). Perceptions of support given by two single item questions	Mother–child dyads (*n* = 226), mothers mean age 75.04 years, children mean age 49.57 years
Woolley 2018 [44]	To explore the perspective of mothers-in-law of interactions with and feelings toward their daughters-in-law, and the son’s role in those interactions.	Social Work/United States	Quantitative	Older mothers (*n* = 267), from various races and religions, who’s sons are married to a living daughter in law. Age mean 63, (SD = 10; range: 37–88)

**Table 2:** Occupations and perceptions within the maternal role identified in the 14 articles.

Instrumental Activities of Daily Living		Social Participation	
Occupations (*n* = 12)	Perceptions (*n* = 7)	Occupations (*n* = 8)	Perceptions (*n* = 5)
Assistance with daily chores [31,37,39,40,41]	Supporting children as part of motherhood [36,39,40,42,43]	Sharing enjoyable activities with children [32,38]	Valuing close relationships [32,37,38]
Providing financial support [36,39,42,43]	Feeling rewarded by supporting children [38]	Open, frequent communication with children [35,40,44]	Close relationships reconcile earlier hardship of motherhood [38]
Caring for children [34,39,43]	Ambivalence towards exchanged support [31,33,35]	Sharing thoughts and opinions with children [33,37]	Viewing children in-law as part of the relationship with children [44]
Caring for grandchildren [38,41]		Visiting children and sharing holiday rituals [31]	
Exchanged support [32,33,35]			

The *n* represents the number of studies.

## Data Availability

Not applicable.

## Appendix A

Preferred Reporting Items for Systematic reviews and Meta-Analyses extension for Scoping Reviews (PRISMA-ScR) Checklist.

**SECTION**	**ITEM**	**PRISMA-ScR CHECKLIST ITEM**	**REPORTED ON PAGE #**
TITLE			
Title	1	Identify the report as a scoping review.	1
ABSTRACT			
Structured summary	2	Provide a structured summary that includes (as applicable): background, objectives, eligibility criteria, sources of evidence, charting methods, results, and conclusions that relate to the review questions and objectives.	1
INTRODUCTION			
Rationale	3	Describe the rationale for the review in the context of what is already known. Explain why the review questions/objectives lend themselves to a scoping review approach.	1–2
Objectives	4	Provide an explicit statement of the questions and objectives being addressed with reference to their key elements (e.g., population or participants, concepts, and context) or other relevant key elements used to conceptualize the review questions and/or objectives.	2
METHODS			
Protocol and registration	5	Indicate whether a review protocol exists; state if and where it can be accessed (e.g., a Web address); and if available, provide registration information, including the registration number.	N/A
Eligibility criteria	6	Specify characteristics of the sources of evidence used as eligibility criteria (e.g., years considered, language, and publication status), and provide a rationale.	2
Information sources	7	Describe all information sources in the search (e.g., databases with dates of coverage and contact with authors to identify additional sources), as well as the date the most recent search was executed.	2–3
Search	8	Present the full electronic search strategy for at least 1 database, including any limits used, such that it could be repeated.	2–3
Selection of sources of evidence	9	State the process for selecting sources of evidence (i.e., screening and eligibility) included in the scoping review.	3
Data charting process	10	Describe the methods of charting data from the included sources of evidence (e.g., calibrated forms or forms that have been tested by the team before their use, and whether data charting was done independently or in duplicate) and any processes for obtaining and confirming data from investigators.	3
Data items	11	List and define all variables for which data were sought and any assumptions and simplifications made.	3
Critical appraisal of individual sources of evidence	12	If done, provide a rationale for conducting a critical appraisal of included sources of evidence; describe the methods used and how this information was used in any data synthesis (if appropriate).	N/A
Synthesis of results	13	Describe the methods of handling and summarizing the data that were charted.	3
RESULTS			
Selection of sources of evidence	14	Give numbers of sources of evidence screened, assessed for eligibility, and included in the review, with reasons for exclusions at each stage, ideally using a flow diagram.	4
Characteristics of sources of evidence	15	For each source of evidence, present characteristics for which data were charted and provide the citations.	5–7
Critical appraisal within sources of evidence	16	If done, present data on critical appraisal of included sources of evidence (see item 12).	N/A
Results of individual sources of evidence	17	For each included source of evidence, present the relevant data that were charted that relate to the review questions and objectives.	7–9
Synthesis of results	18	Summarize and/or present the charting results as they relate to the review questions and objectives.	9
DISCUSSION			
Summary of evidence	19	Summarize the main results (including an overview of concepts, themes, and types of evidence available), link to the review questions and objectives, and consider the relevance to key groups.	9
Limitations	20	Discuss the limitations of the scoping review process.	10
Conclusions	21	Provide a general interpretation of the results with respect to the review questions and objectives, as well as potential implications and/or next steps.	10
FUNDING			
Funding	22	Describe sources of funding for the included sources of evidence, as well as sources of funding for the scoping review. Describe the role of the funders of the scoping review.	11

## References

[1] Beard J.R., Officer A.M., Cassels A.K. (2016). The world report on ageing and health. Gerontologist.

[2] Hocking C., Boyt Schell B.A., Gillen G., Scaffa M.E., Cohn E.S. (2013). Contribution of occupation to health and well-being. Willard & Spackman’s Occupational Therapy.

[3] Martin M., Weibel R., Rocke C., Boker S.M. (2018). Semantic Activity Analytics for Healthy Aging: Challenges and Opportunities. IEEE Pervasive Comput..

[4] Smallfield S., Molitor W.L. (2018). Participation and Leisure Engagement for Community-Dwelling Older Adults: A Systematic Review. Am J Occup Ther..

[5] Stav W.B., Hallenen T., Lane J., Arbesman M. (2012). Systematic Review of Occupational Engagement and Health Outcomes Among Community-Dwelling Older Adults. Am. J. Occup. Ther..

[6] World Health Organization (2017). Global Strategy and Action Plan on Ageing and Health.

[7] WFOT (2013). Definitions of Occupational Therapy.

[8] American Occupational Therapy Association (2014). Occupational Therapy practice framework: Domain & process 3rd Edition. Am. J. Occup. Ther..

[9] Craig B.M., Donovan K.A., Fraenkel L., Watson V., Hawley S., Quinn G.P. (2014). A Generation of Childless Women: Lessons from the United States. Women’s Health Issues.

[10] Monte L.M., Ellis R.R. (2014). Fertility of Women in the United States: 2012. Population Characteristics.

[11] Evans K.L., Millsteed J., Richmond J.E., Falkmer M., Falkmer T., Girdler S.J. (2018). The impact of within and between role experiences on role balance outcomes for working Sandwich Generation Women. Scand. J. Occup. Ther..

[12] Laney E.K., Hall M.E.L., Anderson T.L., Willingham M.M. (2015). Becoming a mother: The influence of motherhood on woman’s identity development. Identity An. Int. J. Theory Res..

[13] Avrech Bar M., Jarus T. (2015). The effect of engagement in everyday occupations, role overload and social support on health and life satisfaction among mothers. Int. J. Environ. Res. Public Health.

[14] Koniak-Griffin D., Logsdon M.C., Hines-Martin V., Turner C.C. (2006). Contemporary mothering in a diverse society. J. Obstet. Gynecol. Neonatal Nurs..

[15] Primeau L. (2000). Divisions of household work, routines, and child care occupations in families. J. Occup. Sci..

[16] Dunbar S.B., Roberts E. (2006). An exploration of mothers’ perceptions regarding mothering occupations and experiences. Occup. Ther. Health Care.

[17] Sethi C. (2019). Mothering as a relational role: Re-evaluating everyday parenting occupations. J. Occup. Sci..

[18] Avrech Bar M., Forwell S., Backman C.L. (2016). Ascribing meaning to occupation: An example from healthy, working mothers. OTJR Occup. Particip. Health.

[19] Dillaway H.E. (2006). Good Mothers Never Wane: Mothering at Menopause. J. Women Aging.

[20] Dunkle R.E., Ingersoll-Dayton B., Chadiha L.A. (2015). Support for and From Aging Mothers Whose Adult Daughters are Seriously Mentally Ill. J. Gerontol. Soc. Work..

[21] Gueta K., Tam S. (2019). Intensive-invisible mothering: The experiences of mothers of adult children with dual diagnosis. Int. J. Ment. Health Nurs..

[22] Kim H.W., Greenberg J.S., Seltzer M.M., Krauss M.W. (2003). The role of coping in maintaining the psychological well-being of mothers of adults with intellectual disability and mental illness. J. Intellect. Disabil. Res..

[23] Poole J.L., Willer K., Mendelson C. (2009). Occupation of Motherhood: Challenges for Mothers With Scleroderma. Am. J. Occup. Ther..

[24] Weitz T., Estes C.L. (2001). Adding Aging and Gender to the Women’s Health Agenda. J. Women Aging.

[25] Peters S.A.E., Woodward M., Jha V., Kennedy S., Norton R. (2016). Women’s health: A new global agenda. BMJ Glob. Health.

[26] Munn Z., Peters M.D.J., Stern C., Tufanaru C., McArthur A., Aromataris E. (2018). Systematic review or scoping review? Guidance for authors when choosing between a systematic or scoping review approach. BMC Med. Res. Methodol..

[27] Arksey H., O’Malley L. (2005). Scoping studies: Towards a methodological framework. Int. J. Soc. Res. Methodol..

[28] Tricco A.C., Lillie E., Zarin W., O’Brien K.K., Colquhoun H., Levac D., Straus S.E. (2018). PRISMA Extension for scoping reviews (PRISMA-ScR): Checklist and explanation. Ann. Intern Med..

[29] Peters M.D., Godfrey C.M., Khalil H., McInerney P., Parker D., Soares C.B. (2015). Guidance for conducting systematic scoping reviews. Int. J. Evid. Based Health.

[30] Levac D., Colquhoun H., O’Brien K.K. (2010). Scoping studies: Advancing the methodology. Implement Sci..

[31] Bromberg E.M. (1983). Mother-Daughter Relationships in Later Life. J. Gerontol. Soc. Work..

[32] Henwood K.L. (1993). Women and later life: The discursive construction of identities within family relationships. J. Aging Stud..

[33] Blieszner R., Usita P.M., Mancini J.A. (1996). Diversity and Dynamics in Late-Life Mother-Daughter Relationships. J. Women Aging.

[34] Francis-Connolly E. (1998). It Never Ends: Mothering as a Lifetime Occupation. Scand. J. Occup. Ther..

[35] Martini T.S., Grusec J.E., Bernardini S.C. (2003). Perceptions of help given to healthy older mothers by adult daughters: Ways of initiating help and types of help given. Int. J. Aging Hum. Dev..

[36] Suitor J.J., Pillemer K., Sechrist J. (2006). Within-Family Differences in Mothers’ Support to Adult Children. J. Gerontol. Ser. B.

[37] Suitor J.J., Sechrist J., Pillemer K. (2007). When Mothers Have Favourites: Conditions under Which Mothers Differentiate among Their Adult Children. Can. J. Aging La Rev. Can. Du Vieil..

[38] Forssén A.S.K., Carlstedt G. (2008). “You Really Do Something Useful with Kids”: Mothering and Experienced Health and Illness in a Group of Elderly Swedish Women. Health Care Women Int..

[39] Schwarz B., Albert I., Trommsdorff G., Zheng G., Shi S., Nelwan P.R. (2010). Intergenerational Support and Life Satisfaction: A Comparison of Chinese, Indonesian, and German Elderly Mothers. J. Cross-Cult. Psychol..

[40] Schwarts Y., Ayalon L. (2015). The experiences of older mothers following the return of an adult child home. J. Aging Stud..

[41] To C.W.-C. (2015). Sibling structure, distributive norms, and negotiation for mothers-in-law’s assistance in rural South China. J. Aging Stud..

[42] Lee Y.-S. (2016). Is giving or receiving psychologically beneficial to older mothers in South Korea? Importance of marital status. J. Women Aging.

[43] Bangerter L.R., Polenick C.A., Zarit S.H., Fingerman K.L. (2018). Life Problems and Perceptions of Giving Support: Implications for Aging Mothers and Middle-Aged Children. J. Fam. Issues.

[44] Woolley M.E., Greif G.L. (2018). Mother-in-Law Reports of Closeness to Daughter-in-Law: The Determinant Triangle with the Son and Husband. Soc. Work.

[45] Francis-Connolly E. (2000). Toward an Understanding of Mothering: A Comparison of Two Motherhood Stages Elizabeth. Am. J. Occup. Ther..

[46] Kielhofner G., Burke J.P. (1980). A Model of Human Occupation, Part Conceptual Framework and Content. Am. J. Occup. Ther..

[47] Kielhofner G., Kielhofner G. (2009). The model of human occupation. Conceptual Foundations of Occupational Therapy Practice.

[48] Shloim N., Hugh-Jones S., Rudolf M.C.J., Feltbower R.G., Lans O., Hetherington M.M. (2015). “It’s like giving him a piece of me.”: Exploring UK and Israeli women’s accounts of motherhood and feeding. Appetite.

[49] Roccas S., Sagiv L. (2009). Personal Values and Behavior: Taking the Cultural Context into Account. Soc. Pers. Psychol. Compass.

[50] Rudnicka E., Napierała P., Podfigurna A., Męczekalski B., Smolarczyk R., Grymowicz M. (2020). The World Health Organization (WHO) approach to healthy ageing. Maturitas.

[51] World Health Organization (2001). ICF: International Classification of Functioning, Disability and Health.

[52] Dawson-Townsend K. (2019). Social participation patterns and their associations with health and well-being for older adults. SSM -Popul. Health.

[53] Lam J., Bolano D. (2019). Social and productive activities and health among partnered older adults: A couple-level analysis. Soc. Sci. Med..

[54] Moieni M., Irwin M.R., Seeman T.E., Robles T.F., Lieberman M.D., Breen E.C., Okimoto S., Lengacher C., Arevalo J.M., Olmstead R. (2020). Feeling needed: Effects of a randomized generativity intervention on well-being and inflammation in older women. Brain Behav. Immun..

[55] Moieni M., E Seeman T., Robles T.F., Lieberman M.D., Okimoto S., Lengacher C., Irwin M.R., Eisenberger N.I. (2021). Generativity and Social Well-Being in Older Women: Expectations Regarding Aging Matter. J. Gerontol. Ser. B.

[56] Yuen H.K., Huang P., Burik J.K., Smith T.G. (2008). Impact of Participating in Volunteer Activities for Residents Living in Long-Term-Care Facilities. Am. J. Occup. Ther..

[57] Gimigliano F., Negrini S. (2017). The World Health Organization “Rehabilitation 2030: A call for action”. Eur. J. Phys. Rehabil. Med..

[58] Cogan A.M., Carlson M. (2017). Deciphering participation: An interpretive synthesis of its meaning and application in rehabilitation. Disabil. Rehabil..

